# The ontogeny of human fetal trabecular bone architecture occurs in a limb-specific manner

**DOI:** 10.1038/s41598-024-67566-w

**Published:** 2024-08-31

**Authors:** Crispin Charles Wiles, Sarah Holly Suh, Katharine Robson Brown, Richard Leslie Abel

**Affiliations:** 1 MSk Laboratory, Sir Michael Uren Hub, Department of Surgery and Cancer, Faculty of Medicine, Imperial College London, London, W12 7ED UK; 2 Centre for Blast Injury Studies, Department of Bioengineering, Faculty of Engineering, Imperial College London, London, SW7 2AZ UK; 3 Warwick Medical School, University of Warwick, Coventry, CV4 8JE UK; 4 Jean Golding Institute for Data Science, University of Bristol, Bristol, BS8 IUU UK; 5 School of Engineering, University of Bristol, Bristol, BS8 1UU UK; 6 Department of Mechanical Engineering, University of Bristol, Bristol, BS8 1UB UK

**Keywords:** Bone, Bone development

## Abstract

Gestational growth and development of bone is an understudied process compared to soft tissues and has implications for lifelong health. This study investigated growth and development of human fetal limb bone trabecular architecture using 3D digital histomorphometry of microcomputed tomography data from the femora and humeri of 35 skeletons (17 female and 18 male) with gestational ages between 4 and 9 months. Ontogenetic data revealed: (i) fetal trabecular architecture is similar between sexes; (ii) the proximal femoral metaphysis is physically larger, with thicker trabeculae and greater bone volume fraction relative to the humerus, but other aspects of trabecular architecture are similar between the bones; (iii) between 4 and 9 months gestation there is no apparent sexual or limb dimorphism in patterns of growth, but the size of the humerus and femur diverges early in development. Additionally, both bones exhibit significant increases in mean trabecular thickness (and for the femur alone, bone volume fraction) but minimal trabecular reorganisation (i.e., no significant changes in degree of anisotropy, connectivity density, or fractal dimension). Overall, these data suggest that in contrast to data from the axial skeleton, prenatal growth of long bones in the limbs is characterised by size increase, without major reorganizational changes in trabecular architecture.

## Introduction

### Background

Bone development is a key process of pre- and postnatal ontogeny, and major abnormalities of this process can significantly impact survival^1^. As a result, determining the mechanisms underlying bone development is an important task in biology and medicine. Yet while much progress has been made on unravelling the molecular basis of bone development^2,3^, the development of the material and structural features that contribute to mechanical properties of fetal bones remain understudied.

Furthermore, there is increasing recognition that developmental health can impact adult bone disease susceptibility^4,5^. For example, a recent study showed fetal genetic and maternal intrauterine contributions to birthweight causally influence future adult bone mineral density (BMD)^6^, and others have suggested early bone health affects adult morbidity, such as risk of osteoporosis and bone fracture^7,8^. These findings emphasise the need to understand how the mass and architecture of fetal bone develops over time. Whilst several studies have examined changes in trabecular bone occurring from birth onwards, for example in the humerus and femur^9–11^, few studies have examined the gestational time period in any detail, in part because the paucity of fetal skeletal material makes such studies challenging to conduct, especially with human tissue^12–14^.

### 2D analyses of fetal bone

Most early studies of fetal bone development employed 2D approaches, using either conventional microscopy or microcomputed tomography (µCT). The histological development of the fetal humerus^15^ and femur^16^ has been described qualitatively in detail, and measurements of overall growth have also been reported (see ^17^ for details). More recent quantitative histological work focusing on trabecular bone has found that bone volume fraction (BV/TV) increases in the proximal femur between 16 and 41 weeks’ gestation^18,19^. This was attributed to increases in mean trabecular thickness (Tb.Th), rather than increases in number of trabeculae (Tb.N)^19^. In contrast, later work using low resolution 2D µCT images found minimal change in BV/TV during this period of development^13^. Whilst increases in Tb.Th between 16 and 40 weeks’ gestation were also observed in this study, a concomitant decrease in Tb.N resulted in no overall change in BV/TV.

Although 2D µCT data has been reported as representative of histological measurements of bone trabecular structure^20^, the disagreement in results between these studies may be related to the sampling and measurement methods. Furthermore, 2D measurement of inherently 3D parameters such as BV/TV are only approximations (typically determined by the proportion of bone relative to the total length of a line transecting the bone on a 2D section).

Clarifying whether BV/TV changes during gestation has potential implications for our understanding of the significance of the fetal period for lifelong bone health, as BV/TV is known to be a major determinant of the mechanical properties of bone^21,22^.

### 3D µCT analyses of fetal bone

The most effective approach to image, sample and measure the complex 3D architecture of trabecular bone in a non-destructive manner is using high-resolution 3D µCT^23,24^. An early study employing these methods to study vertebrae reported an increase in BV/TV between 16 and 24 weeks gestation, but found no change in Tb.Th throughout gestation^25^. A more comprehensive study of vertebrae confirmed this increase in BV/TV during gestation, between 26 and 40 weeks^14^. The same study also reported complex and dynamic changes in Tb.Th and other features of trabecular architectural organisation during both gestation and early infancy. These included increased Tb.N, increased connectivity density (Conn.D), decreased degree of anisotropy (DA), and a shift to more plate-like trabecular morphology (based on measurement of structural model index, SMI) up to birth, followed by a drop in most measures postnatally. These data were interpreted as initial “overproduction” of bone during gestation, followed by reductions in BV/TV, Tb.Th and Tb.N in the year after birth, which the authors described as postnatal “sculpting” of trabecular architecture, with further “refinement” in later infancy^14^.

A similar process of post-natal trabecular “sculpting” during early infancy has been observed for various appendicular skeletal bones from humans^26,27^, Neanderthals^27^, and non-human primates^28^, with a reduction in BV/TV and Tb.N. However, in contrast to the findings in the vertebrae outlined above, this was accompanied by a significant increases in Tb.Th with age in the appendicular bones examined. Although these studies of the appendicular skeleton focus on postnatal changes, their findings highlight the potential for regionally-specific changes in trabecular architecture, which may relate to function. Similarly, some authors have identified the development of regional differences in trabecular architecture within a single bone. For example, the new-born ilium has been hypothesised to anticipate future functional demands^12^, based on the presence of a pattern of trabecular architecture at birth that appears to resemble that required by the loading demands of bipedal locomotion.

To summarise, the few studies on fetal trabecular bone development broadly agree that the prenatal period is characterised by rapid bone production, perhaps for modelling postnatally when the skeleton is subject to increased postural and locomotor loads. However, more high-quality 3D data from the gestation period are needed, particularly for the appendicular skeleton, where the limited number of studies have reported markedly different findings.

### Aims and objectives

The aim of this study was to investigate the growth and development of trabecular architecture through gestation using high-resolution 3D µCT scans of the femora and humeri in 35 skeletons (17 female and 18 male) of gestational age ranging between 4 and 9 months. The specific objectives were to compare trabecular architecture between (i) females and males; (ii) proximal humeral and femoral metaphyses; and (iii) fetal age categories (from 4 to 9 months gestation). The cross-sectional data were analysed to improve our understanding of prenatal development in trabecular architecture in the appendicular skeleton.

## Results

**Figure 1:**
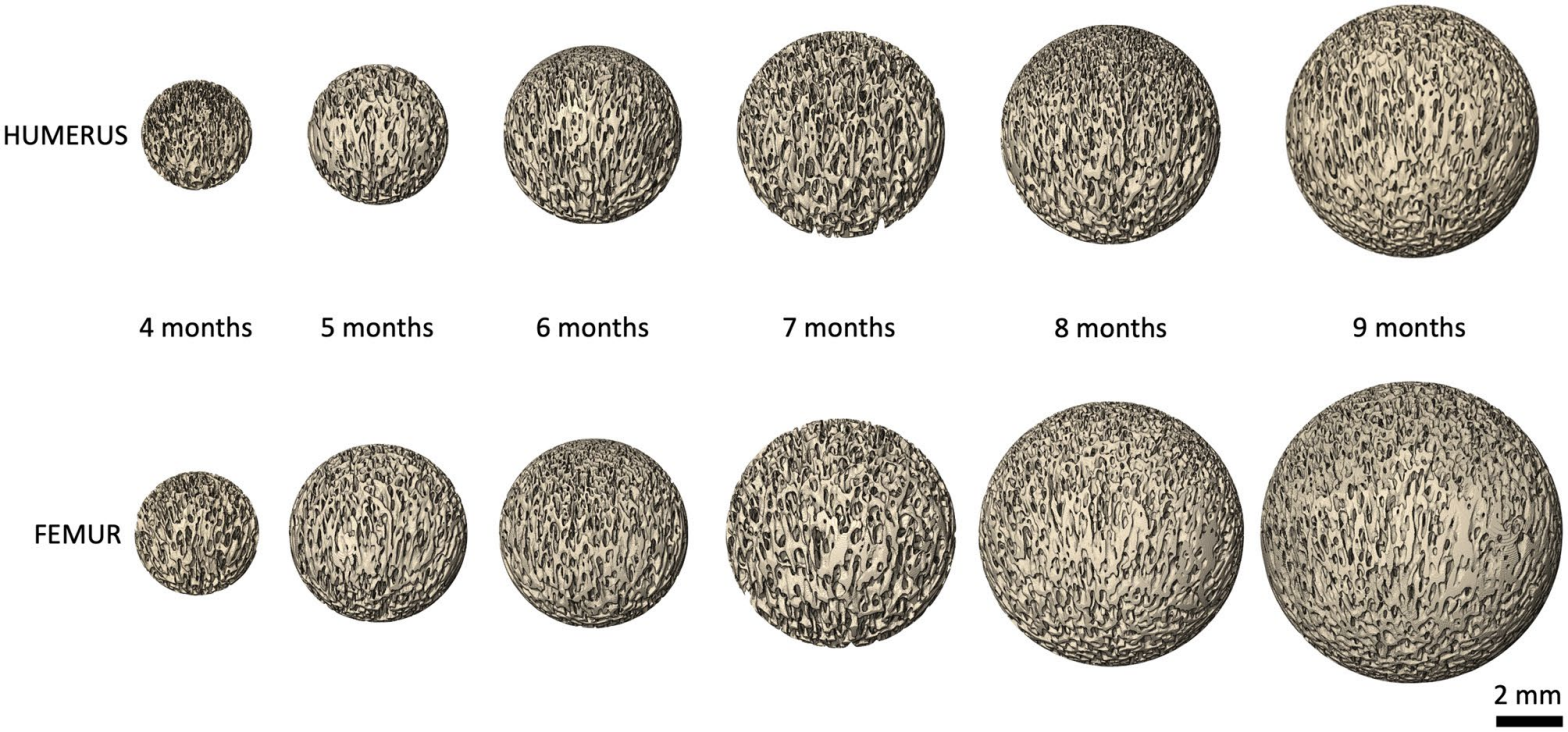
Representative spheres sampled from homologous regions of trabecular bone from the proximal metaphyses of fetal humeri and femora, aged 4–9 months gestation. Spheres are 3D volumetric reconstructions of µCT data (isotropic voxel size: 10 µm), shown in orthographic camera projection for comparability of scale. Scale bar = 2 mm.

Raw data showing all results from digital histomorphometry for each Volume of Interest (VOI) from 35 humeri and 35 femora (gestational age 4–9 months) are shown in Tables S1 and S2 respectively. VOIs representing each fetal age bracket were volumetrically rendered in orthographic camera projection for both humerus and femur to aid in visual comparison (Fig. 1).

### Sex comparisons

**Figure 2:**
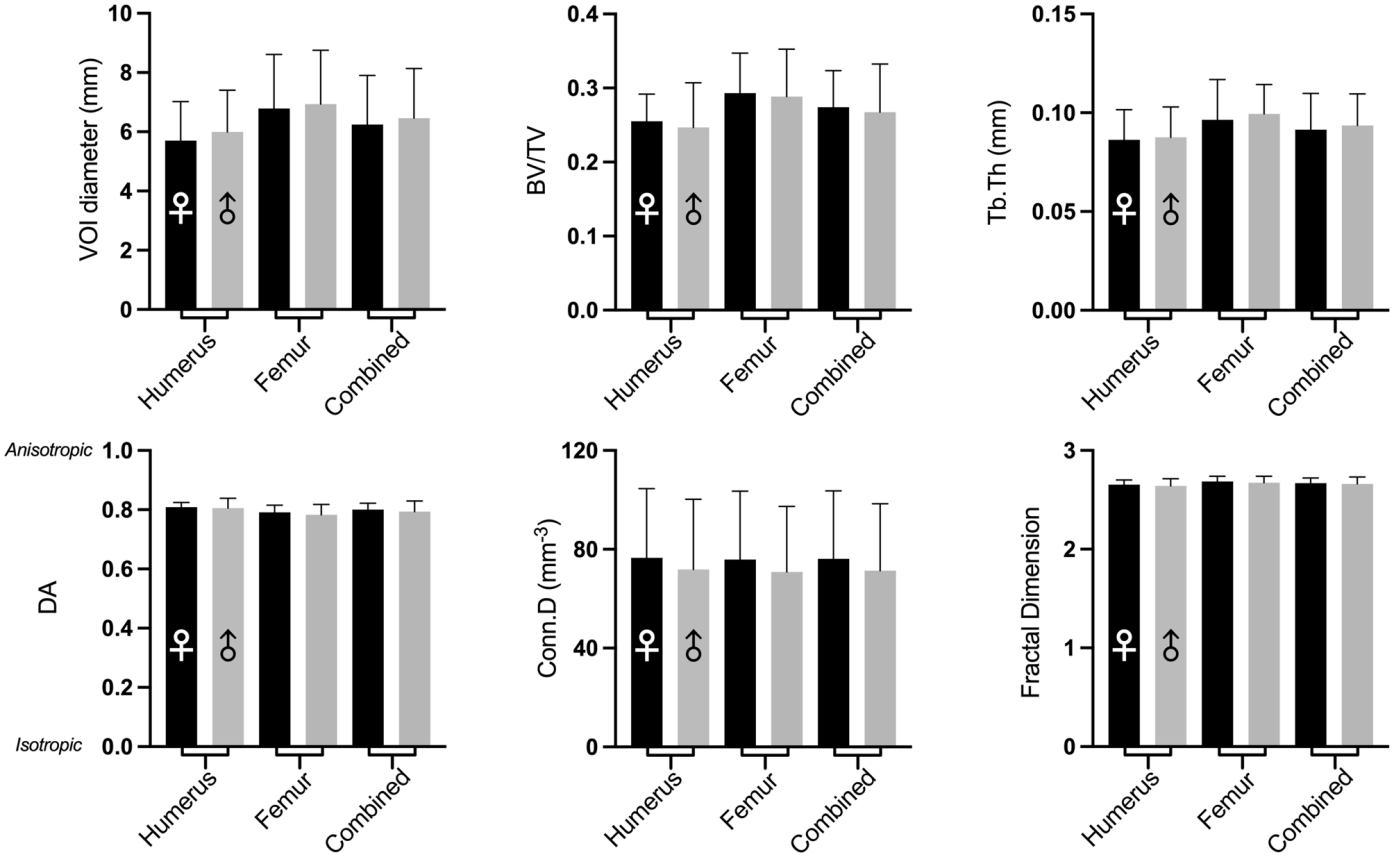
Mean and standard deviation of fetal trabecular architectural parameters for females (black bars) and males (grey bars). Comparison reveals trabecular architecture of fetal proximal limb bones is similar in both sexes, with no apparent sexual dimorphism. Data were compared by one-way ANOVA with Šídák *posthoc* tests (only shown for VOI diameter). There were no significant comparisons (ns = *p* > 0.05).

When data from all fetal ages were pooled, females (n = 17) and males (n = 18) exhibited similar trabecular architecture in both the humerus and the femur (Fig. 2 and Table S3 for details). Similarly, no sex differences were observed when humeral and femoral data were combined to increase statistical power (one-way ANOVA *p* > 0.05).

### Upper and lower limb bone comparisons

**Figure 3:**
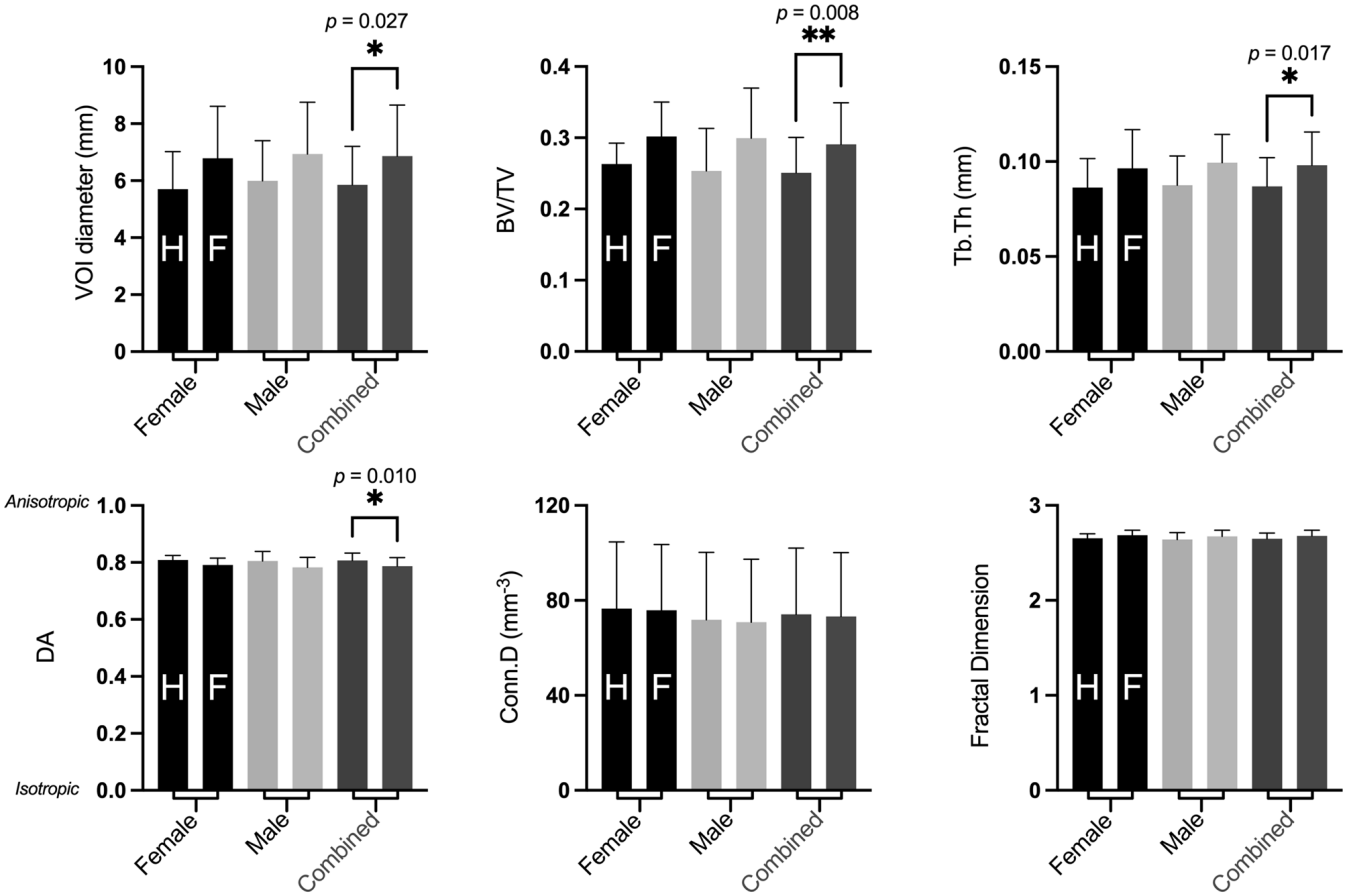
Mean and standard deviation of fetal trabecular architectural parameters for the humerus (left bar in each pair) and femur (right bar in each pair) in females (black bars), males (light grey bars), and female and male data combined (dark grey bars). Comparison of combined male and female datasets revealed the fetal femur is larger (VOI diameter), with increased bone volume fraction (BV/TV) and trabecular thickness (Tb.Th) than the fetal humerus and exhibited slightly lower DA. Data were compared by one-way ANOVA with Šídák *posthoc* tests. *P* values shown for significant results from one-way ANOVA. Asterisks indicate significant differences in *posthoc* testing: *p* < 0.05 (*) and *p* < 0.01 (**).

When data from all fetal ages were pooled, comparison of trabecular architecture between humerus (n = 35) and femur (n = 35) using one-way ANOVA did not reveal significant differences when male and female data were analysed independently (Fig. 3 and Table S4 for details). However, apparent trends were visible, with VOI diameter, BV/TV and Tb.Th appearing greater in the femur, and DA appearing greater in the humerus. These apparent trends in humeral *vs* femoral trabecular architecture became statistically significant when the male and female datasets were combined to increase statistical power (VOI diameter: F = 2.868, *p* = 0.017, BV/TV: F = 3.774, *p* = 0.003, Tb.Th: F = 3.240, *p* = 0.009, and DA: F = 3.728, *p* = 0.003).

### Gestational age comparisons

**Figure 4:**
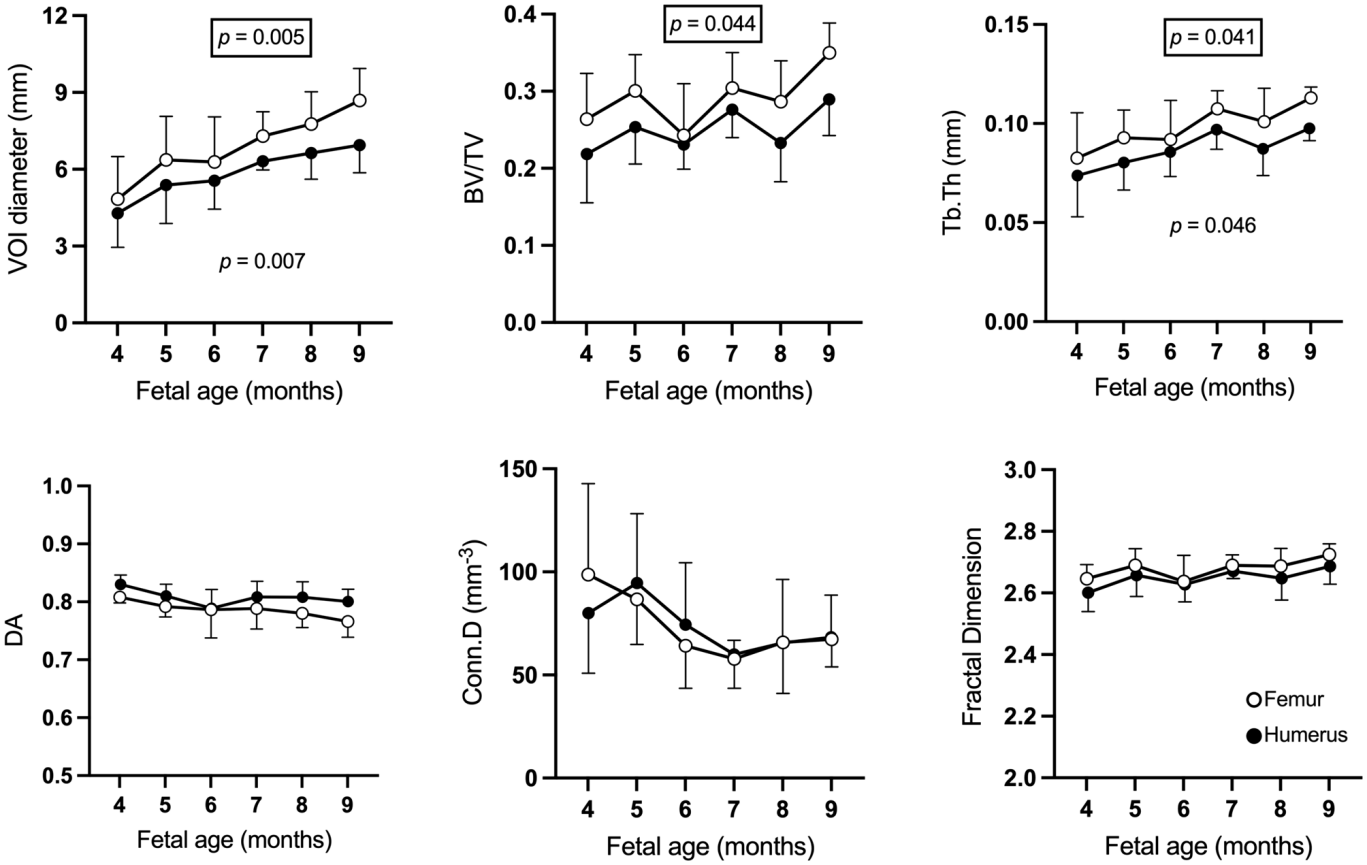
Mean and standard deviation of fetal trabecular architectural parameters at different fetal ages for humerus (filled circles) and femur (empty circles). Male and female data have been pooled. Comparison of mean morphometric values at 5–9 months relative to 4 months reveals significant increases in the size of the femur and humerus (VOI diameter), trabecular thickness, and BV/TV (femur only) during gestation. However, little reorganisation of trabecular structure is observed in either bone. Data were compared by one-way ANOVA with Šídák *posthoc* tests. *P* values shown for significant results from one-way ANOVA (unboxed = femur, boxed = humerus). Details of *P* values from Šídák *posthoc* tests can be found in Table S5.

To examine for changes related to fetal age, male and female data were pooled on the basis that no significant sex differences had been observed. For each measure of trabecular architecture, mean values at 4–9 months were plotted, which suggested VOI diameter, BV/TV, and Tb.Th all gradually increased with increasing fetal age. To test if this increase was statistically significant, mean values at 4 months were compared to mean values at 5, 6, 7, 8, and 9 months using one-way ANOVA (see Fig. 4 and Table S5 for details). For both the humerus and the femur, bone size increased significantly during gestation (humeral VOI diameter: F = 3.961, *p* = 0.007; femoral VOI diameter: F = 4.348, *p* = 0.005). This is shown visually by the increasing size of VOIs shown in Fig. 1 (also see Figure S2). In addition, both bones also exhibited significant increases in Tb.Th during gestation (humeral Tb.Th: F = 2.601, *p* = 0.046; femoral Tb.Th: F = 2.692 *p* = 0.041). *Posthoc* comparisons relative to Tb.Th values at 4 months indicate these changes were all significant by 9 months gestation (see Table S5 for further details). BV/TV also increased with gestational age. This reached significance on one-way ANOVA for the femur (F = 2.645, *p* = 0.044) due to a significant difference between 6 and 9 months, but not the humerus (F = 1.913, *p* = 0.123). Note that the standard deviation for the femur at six months is somewhat greater than at other time points. Formal *posthoc* comparisons relative to BV/TV values at 4 months indicated an increase at or near trend-level for both femur and humerus by 9 months gestation.

## Discussion

The present study collected high resolution 3D µCT data from 70 fetal bones (35 humeri and 35 femora) from an ontogenetic series (4–9 months gestation) and performed digital histomorphometry on the trabecular architecture. This represents the first attempt to test hypotheses on the effects of sex, limb, and gestational age on human fetal trabecular architecture in limb bones using high-resolution 3D methods. Together, these data reveal that the sex differences in trabecular architecture of the limb bones seen in adult life are not established prenatally. The data also show that the proximal fetal femoral metaphysis is larger (i.e. accommodates a VOI of greater diameter) compared to the humerus during this period of gestation, with a higher bone volume fraction (BV/TV) and mean trabecular thickness (Tb.Th), but a marginally lower degree of anisotropy (DA). Finally, as the proximal metaphyses grow during gestation (as shown by increasing VOI), the trabeculae of the humerus and femur become progressively thicker (increased Tb.Th) with a trend towards (humerus) or significant (femur) increases in bone volume fraction (BV/TV). Yet there is minimal trabecular reorganisation during gestation i.e., no significant change in degree of anisotropy (DA), connectivity density (Conn.D), or fractal dimension (FD). This pattern of long bone growth and development (increase in size, Tb.Th and, for the femur, BV/TV, with only minor reorganisation of trabecular architecture) during the fetal period clarify conflicting findings from previous 2D studies of the humerus^13^ and femur^13,18,19^, and contrast with 3D reports of trabecular development in other anatomical sites, e.g. the vertebrae^14^. Together, the data suggest that ontogeny of fetal trabecular architecture proceeds in a limb-specific and even bone-specific manner.

### Fetal trabecular bone is not sexually dimorphic

No fetal sex differences in bone size (based on VOI diameter) or any measures of fetal trabecular architecture of the proximal humeral and femoral metaphyses were observed when pooled samples of all gestational ages were compared (Fig. 2). Comparisons at each gestational age were not performed due to limited sample size.

Whilst the finding of no sexual dimorphism of bone size for the femur and humerus is limited to data on the diameter of the maximal sphere to fit into the fetal metaphyses (VOI), this interpretation is broadly supported by ultrasound measurements of whole femoral length^29^ (although some studies have reported minor sex differences^30^), and radiological measurements on other metrics of bone size including diaphyseal diameter and cortical area^31^.

The finding of no sexual dimorphism in fetal trabecular architecture confirms the results of a previous 2D µCT study^13^, which tested for sexual dimorphism at each gestational age. However, there is a marked discrepancy in the absolute measurements of some trabecular parameters between the studies. For example, mean femoral Tb.Th. measurements at 9 months gestation were approximately fourfold greater in the 2D µCT study than those in the current study (around 0.41 mm *vs* 0.11 mm). As recognised by the authors of the 2D µCT study, both voxel size and 2D measurement methodology influence measurement. For instance, the Nyquist-Shannon sampling theorem states a signal (i.e. the width of a trabecula) must be sampled by at least twice the bandwidth of the signal to allow accurate characterisation^32^. As the high-resolution scan data (isotropic voxel size = 10 µm) and 3D measurements of the current study absolutely resemble those from independent 2D histological reports^18,19^, it is likely that the relatively large voxel size (120 × 120 × 100 µm) used in the 2D µCT study^13^ systematically biased the measurements e.g., by missing finer trabeculae. Under-sampling of these finer trabeculae would skew average Tb.Th measurements, resulting in exaggerated Tb.Th values. This issue is likely to have been especially problematic in the area immediately distal to the growth plate, where trabeculae are particularly thin (see Fig. 1 and^19^). Whilst low resolution leads to underestimation of absolute measures of Conn.D and overestimation of Tb.Th and BV/TV (see^27^ for examples of quantitative impact of low resolution on 3D morphological measurements), the overall conclusion of no evidence of fetal trabecular sexual dimorphism is shared by both 2D and 3D µCT studies.

In contrast, postnatal sexual dimorphism in trabecular bone is well known, emerging during puberty^33^ and continuing post-menopause^34^. This is thought to be driven by numerous factors that vary with sex, including metabolism and endocrine environment^21,35^. For example, testosterone and estradiol significantly impact bone mineral homeostasis in adults^36^. Interestingly, these factors are also known to exert developmental programming effects that influence later life, such as likelihood of metabolic disease and neural structure/function^37^. For example, at term, the birthweight of male new-borns is typically greater than that of female new-borns, which may imply that fetal growth begins earlier in males^38^ and increased female fetal testosterone levels are associated with greater gestational weight gain^39^, suggesting a potential role for endocrine signalling to influence the gestational environment and growth.

This raises the questions why sexually dimorphic trabecular architecture is not observed prenatally and when is it established? One potential explanation for the lack of sexual dimorphism during gestation is that fetal sex hormones generally circulate at relatively low levels during gestation. Only minor and temporary increases in male fetal testosterone levels are seen during gestational weeks 11–18^40^. By term, both testosterone and estradiol levels are approximately similar in both male and female neonates and are markedly lower than maternal concentrations^40^. Whilst limited, these and other data (including normal birthweight and growth observed in a baby lacking the alpha estradiol receptor^41^) indirectly suggest these hormones have a very limited impact on skeletal development in utero. In contrast, data from knock-out mice indicate that non sex-specific hormones such as parathyroid hormone, and in particular parathyroid hormone-related peptide, exert a powerful influence on fetal skeletal morphogenesis, affecting both cortical and trabecular regions of fetal bone^36,42^.

Given the lack of sexual dimorphism observed in fetal trabecular bone, it is of interest that sexual differences in intrauterine limb movements have been reported^43^, especially given the role of mechanical loading in shaping adult trabecular architecture is well-recognised^21,23,44,45^. One hypothesis that may account for this apparent discrepancy relates to the mechanostat concept, which suggests mechanical bone strains, e.g. generated by muscle forces, only influence bone morphology above certain thresholds^46–48^. If this is correct, the reported sex differences in the patterns of fetal movement during gestation may be insufficient to result in differential trabecular morphology. The significance of these movements is further discussed below in reference to upper *vs* lower limb differences.

Further experiments are required to test if the apparent lack of sexual dimorphism in fetal trabecular architecture reported here is confined to the femur and humerus or reflects a general pattern throughout the skeleton, and to confirm when in ontogeny sexual dimorphism of trabecular bone appears.

### Fetal trabeculae architecture differs between femur and humerus

Limb-specific differences in trabecular architecture were observed between the proximal femoral *versus* humeral metaphysis when male and female data were pooled (Fig. 3). The femur exhibited significantly greater size (shown by VOI), with a higher bone volume fraction (BV/TV), thicker trabeculae (Tb.Th) and slightly reduced anisotropy (DA) relative to the humerus. This contrasts with the findings from the 2D µCT study of fetal bone discussed above, which reported no differences in trabecular measures between fetal humeri and femora^13^. Aside from the methodological issues outlined above, this discrepancy may reflect different approaches to comparison. The 2D µCT study compared relatively small numbers of humeral and femoral samples at each gestational age. Similarly, the paucity of perinatal specimens may explain why other studies focusing on postnatal ontogeny have not observed these limb-specific trabecular differences at birth, but have identified a divergence in trabecular architecture between humerus and femur from around 1 year after birth, in response to the mechanical demands associated with the development of limb-specific functional specialisation during the acquisition of bipedal walking^9,10,49^. In the current study, significant differences were not observed when data from all age groups were pooled, and were only seen when both male and female data were also combined (see Fig. 3). This suggests the limb-specific trabecular differences between humerus and femur occurring during gestation are relatively modest and so require greater statistical power to reveal them.

Two potential explanations for the limb-specific differences in fetal bone size and trabecular architecture observed in this study include: (i) differences in the timing of the formation of the cartilage precursor (anlage) or primary ossification centre for each bone; and/or (ii) limb-specific differences in bone loading.

In terms of gestational timings, chondrification for both femur and humerus is complete by the end of the 8th embryonic week, and the primary ossification centre appears by weeks 8–9^17^. As a result, the relatively increased size, trabecular thickness and bone volume fraction of the femur cannot simply be attributed to an earlier onset of development but may reflect a larger cartilage anlage for the femur. Similarly, given the trabecular organisation of the primary spongiosa formed early in development has been argued to reflect the calcification and subsequent endochondral ossification patterns of the cartilage template established in chondrification^49,50^, structural differences in anlagen could also underlie the differences observed in the pattern of humeral *vs* femoral trabecular bone. This hypothesis is further discussed below in relation to the persistently high degree of anisotropy observed in both bones throughout gestation.

Limb-specific differences in mechanical loading is another potential driver of limb-specific differences in trabecular architecture. First, mechanical loading is known to influence bone morphology and trabecular architecture postnatally ^21,23,44,45^. In addition, studies have increasingly highlighted the importance of mechanical cues in regulation of key skeletal developmental pathways^21,51,52^. These, in conjunction with the appropriate morphogenetic cues, appear to be key for driving mesenchymal stem cell differentiation that influences both bone development^53^ and repair^54^ and ultimately affects bone structure.

It is therefore plausible that intrauterine fetal movements occurring as a normal part of development may influence fetal trabecular bone development. Fetal movements usually commence by around 10 weeks’ gestation and continue until term^55,56^. The importance of such movements for overall bone development is highlighted by the thin, hypomineralised long bones observed in neonates who have experienced fetal immobility due to neuromuscular disease^57^. The relevance of fetal movement on bone development is further supported by indirect evidence that fetal position in utero influences bone mass, independent of size at delivery. For example, breech presentation is associated with reduced bone mass and area (based on dual-energy X-ray absorptiometry measurements), which has been hypothesised to be due to reduced fetal movement^58^. However, no data on position in utero was available for the samples used in this study. Finally, modelling of fetal movements suggests the intramuscular forces experienced by muscles crossing different joints differ significantly^59^, which could contribute to limb and site-specific differences in bone loading.

Ultimately, hypotheses on the effects of fetal movement on fetal bone architecture remain relatively untested, and further details about the differential direction and magnitude of loading experienced by the humerus and femur in utero are required to test the hypothesis that site-specific loading differences drive the increased femoral bone volume fraction and trabecular thickness observed in this study. Imaging techniques such as 4D MRI or quantitative ultrasound may provide routes by which site-specific movement and measures of bone quality could be directly measured and followed up longitudinally to better test these hypotheses.

### Gestation is characterised by fetal limb growth without trabecular reorganisation

Between 4 to 9 months gestation the main changes observed in the proximal metaphysis of both humerus and femur were bone growth (increases in VOI) (Fig. 1) and increased trabecular thickness (Tb.Th). Both humerus and femur showed an apparent increase in BV/TV with increased gestational age (see Fig. 4), but this only reached significance in one-way ANOVA testing for the femur. Minimal reorganisation of the trabecular architecture was observed, i.e., there were no significant changes in degree of anisotropy (DA), connectivity density (Conn.D), or fractal dimension (FD) (see Fig. 4).

The increase in femoral BV/TV reported here was largely due to a 25% increase in Tb.Th between 4- and 9-months gestation. No significant changes in Conn.D. were observed over this period. This contrasts with a prior 2D µCT study which reported no increase in BV/TV for either femur or humerus over the same gestational period^13^. In this prior study, although both bones exhibited approximately 100% increase in Tb.Th between 4 and 9 months gestation, the impact of this on BV/TV was negated by a concomitant 50% reduction in Tb.N^13^. However, in addition to the methodological limitations of this 2D study discussed above, two other strands of evidence support the findings of an increase in femoral BV/TV observed in the current study. First, whilst Conn.D and Tb.N are not directly comparable measures, the major reduction in Tb.N reported in the 2D µCT study would likely result in a significant decrease in Conn.D. However, this was not observed in the current study, suggesting that if a reduction of Tb.N does occur, it is relatively subtle (the rationale for not performing Tb.N measurements in this study is outlined in the Methods). Second, earlier 2D histological studies over a similar period of gestation also reported increases in femoral BV/TV and Tb.Th without significant changes in Tb.N, and at a similar extent to the data presented in the current work^18,19^.

### Explanations for the ontogenetic trajectory of fetal trabecular architecture

Overall, the changes in trabecular structure reported here for fetal humerus and femur show some differences and some similarities compared to prior work on elements of the fetal axial skeleton (specifically vertebrae) and pelvic girdle (specifically iliac bones) reported elsewhere.

Similar to the humerus and femur, fetal vertebral trabecular architecture is broadly characterised by increasing BV/TV and Tb.Th during gestation (6 months to term examined)^14^. Because overall bone mineral density^60^, vertebral BV/TV and Tb.Th^14^ subsequently decline over the year following birth, the prenatal period has been described as representing an “overproduction” of vertebral trabecular bone, which is hypothesised to represent the formation of a calcium reservoir to sustain future growth^14^ in the postnatal period when maternal calcium levels in breast milk are outstripped by infant demand^61^. The interpretation of fetal “overproduction” of bone in the femur and humerus cannot be definitively determined in the current study as no postnatal samples were examined. However, another high-resolution 3D µCT study examining postnatal femoral ontogeny reported BV/TV values in neonates that were approximately equivalent to the measurements reported here at 9 months gestation, and which subsequently declined over the first postnatal year^11^. This provides limited support for the interpretation of a modest “overproduction” of fetal trabecular bone, at least in the femur, with subsequent postnatal loss, although caution is required when attempting comparison of BV/TV measurements between studies, as experimental details such as variation in voxel size, the location of the VOI, and image processing can significantly affect absolute measures.

Interestingly, unlike what is seen in the fetal humerus and femur, in the fetal vertebrae some measures of trabeculae structure suggest significant architectural reorganisation (increased in Tb.N, Conn.D and a decrease in DA) between 6 and 9 months gestation^14^. It is currently unclear whether these reorganizational changes in vertebral trabecular architecture relative to the humerus and femur reflect different responsiveness to localised, dynamic changes in the intrauterine environment or mechanically or genetically programmed differences in bone development. In contrast, postnatal ontogenetic changes in some measures of trabecular architecture reported elsewhere appear broadly similar between limb and axial skeletal elements described.

Postnatally, vertebral trabeculae undergo considerable architectural “sculpting” with a decrease in BV/TV, Tb.Th, Conn.D, and Tb.N, and an increase in DA by 1.2 years after birth, followed by further “refinement”^14^. Other studies suggests that a broadly similar “sculpting” process occurs in the proximal femur, with a postnatal decrease in BV/TV and Tb.N, although Tb.Th tends to increase rather than decrease^9,11^. A similar pattern has also been observed postnatally for the humerus^27^ and calcaneus^26^ (although Tb.N was not reported in this latter study). Whilst again caution is needed when making inter-study comparisons, overall, a rapid postnatal decline in BV/TV in all these bones is broadly in line with the calcium reservoir hypothesis outlined for the vertebrae above.

This is relevant in the context of the current study on fetal bone as it has been suggested that in vertebrae, the trabecular BV/TV values seen at birth not only drop rapidly postnatally but never recover to term levels^14^. This implies the fetal period may be a key time window for maximising BV/TV, with implications for adult bone health. However, the pattern of trabecular architectural changes seen in the appendicular skeleton is complex and BV/TV is highly variable, both between bones and regionally within them. For example, some ontogenetic studies broadly support the above conclusion, noting that adult trabecular BV/TV does not fully return to levels seen at term, e.g., in the proximal humerus^27,49^ and proximal tibia^10,27,49^. However, for the proximal femur, the data differ between studies^9,11^, and in the calcaneus, recovery of BV/TV seems to vary across different functional regions^26^ (for summary of these data see Table 6 in ^27^). Ultimately, the absence of postnatal data in the current study means it is not possible to draw robust conclusions on the recovery (or otherwise) of BV/TV after birth in the humerus or femur. Larger standardised studies charting the full ontogenetic sequence from early gestation to old age are required to fully address these issues.

Fetal ontogeny of trabecular architecture for the humerus and femur from the current study also differs from that reported for the iliac bones. A 2D study examining trabecular architecture within the neonatal ilium reported marked regional variation within the bone, with some areas apparently showing a trabecular alignment that anticipated the adult configuration^12^. The authors interpreted this finding as evidence for a non-mechanical driver of trabecular architecture, and postulated the presence of a site-specific, predetermined template (possibly also influenced by intra-uterine reflexive limb movements), which is later refined postnatally during the development of bipedal locomotion.

Given that postnatally, high DA is often associated with a consistent loading direction^45,62^, it is interesting that in the proximal metaphysis of both the humerus and femur, the absolute DA measurements vary little and remain high throughout gestation. Whilst absolute DA values are influenced by method of calculation^63^, the values reported here in the limb bones are almost 200% of those seen in fetal vertebrae^14^ obtained using the same method. Could this sustained DA represent a similar fetal anticipatory adoption of the adult trabecular configuration? This seems unlikely, as the absolute DA values seen in the fetal bones are even higher (10–20%) than those obtained in similar regions of the adult femur using the same measurement technique^64^. In addition, the adult proximal femoral metaphysis (i.e. the femoral neck) is characterised by two different trabecular arcades, one oriented horizontally and the other vertically^65^. This is not apparent in the fetal period based on visual inspection of the imaging data, and would likely result in lower DA values than those observed in the current study, which indicate a highly consistent primary trabecular orientation. Furthermore, postnatally DA (determined using a different method of measurement) in the proximal femoral metaphysis declines between birth and age 1–2, so there is no obvious persistence of the high DA into adulthood^9^.

Histological data suggest that changes in trabecular morphology in the femur during the fetal period are due to a modelling (rather than remodelling) process, based on the relative speed of trabecular thickening during the gestational period^19^. Whilst modelling is often driven by mechanical stimuli, this seems unlikely to be the basis of the persistent high DA observed throughout the fetal period for two reasons. First, there is little change in DA throughout gestation, despite predictable changes in type and intensity of fetal movements^66^. Second, patterns of fetal movements are known to be sexually dimorphic^43^, but do not lead to sexually dimorphic trabecular architecture.

Therefore on current evidence, a more plausible explanation for the high DA in the fetal humerus and femur is that it reflects the prior anisotropic organisation of the underlying cartilage models, namely the columnar arrangement of cartilage formed during chondrification^50^. These subsequently undergo mineralisation and ossification during the formation of the primary spongiosa^67^. Site-specific variation in cartilage structure and mineralisation could therefore contribute to the inter-limb differences observed between humerus and femur in the current study. However, the causes of such variation are unclear and are likely multifactorial, shaped by genetic, mechanical, and other cues^54^.

Interestingly, some recent studies have reported evidence for an underlying topological blueprint for trabecular bone, where trabecular architecture is analysed as a network, based on nodes (where trabeculae join) and edges (straight lines representing trabeculae)^68^. In contrast to the hypothesised site-specific blueprint for the ilium described earlier^12^, this topological blueprint appears to be site-independent^69^, and persists in the face of major trabecular architectural reorganisation following changes in loading direction^70^. If this is correct, then a similar trabecular topology should be identifiable in fetal bone, and may relate to the topology of the underlying cartilage model or patterns of ossification. However, the presence of this topological organisation in fetal bone has not yet been demonstrated.

### Limitations and further studies

There are some important limitations to this study. First, the cross-sectional study design means interpretations of trabecular changes during gestation are inferred rather than directly observed longitudinally. Second, the gestational age of the specimens is based on maternal testimony, and the specimens come from historical stillbirths where the health of the population could compromise growth and development of the sampled cohort. Furthermore, there is some inter-individual variation in VOI diameter within each gestational age bracket (see Tables S1 and S2), which can potentially be influenced by several factors including nutritional status, inaccuracy of maternal testimony of age, anatomical variation, and discretizing fetal age into monthly intervals. However, while there is no direct data available on the nutritional status of this historical collection, it has been shown previously that the ontogenetic trajectory in this collection and modern populations is similar, and maternal testimony correlates relatively well with other proxies of gestational age such as bone length^13^. Furthermore, Fig. 4 suggests the amount of inter-sample variation in VOI diameter is broadly similar at all fetal age groups, and there remains a clear, progressive increase in VOI diameter at the group level across each fetal age, which eventually becomes statistically significant. If maternal malnutrition or error in maternal testimony is affecting the data, the effect is not significant enough to obscure this pattern of growth. Linked to this, in should be noted that in general standard deviations for most morphological measurements vary considerably between individuals in the same age bracket. Marked inter-individual variation is often observed in ontogenetic analyses of trabecular morphology but future students exploring the effects of sample size and development would be of value. Third, the analysis was limited to a sphere collected from the proximal metaphysis of each bone. Whilst consistent, as a result, only a proportion of the fetal trabeculae have been measured, and region variation within the bone may have been missed. Finally, the data reported are observational, so the significance of the findings are inferences rather than direct tests of causality. Tractable experimental models are required to test directly many of the specific hypotheses generated by such work.

As a unique source of data on fetal trabeculae ontogeny, this study raises multiple questions, some of which have been highlighted above. In addition, furthers studies using these specimens are planned to examine the mechanical properties of the cortical bone and address the issue of limited sampling by examining fetal trabecular architecture using a recently developed whole-bone approach^71^. This will allow a more comprehensive evaluation of the functional ontogeny of the fetal limb bones. In addition, a study using these fetal specimens to test for the presence of the topological blueprint in early ontogeny is currently underway.

## Conclusions

For the limb bones (femur and humerus) studied, gestation is mainly about growth in size without reorganisation of the trabecular architecture. This differs from the changes in trabecular architecture reported for fetal vertebrae and the anticipatory changes reported for the iliac bones by birth. The major findings are: (i) there is no apparent sexual dimorphism in humeral or femoral trabecular architecture between 4 and 9 months gestation, suggesting the dimorphism seen in adults is only established postnatally; (ii) the proximal metaphysis of the fetal femur is relatively larger compared to that of the fetal humerus, with thicker trabeculae, a higher bone volume fraction, and slightly lower anisotropy; and (iii) during gestation, bone size and trabecular thickness gradually increase for both femur and humerus. In addition, there is a trend for increase in BV/TV, although this was only statistically significant for the femur. However, in contrast to reports on fetal vertebrae, minimal reorganisation of trabecular architecture is seen in either bone during gestation. This pattern of development suggests that ontogeny of fetal trabecular architecture occurs in a limb-specific manner.

## Methods

The ontogeny of trabecular architecture in the proximal femur and humerus was analysed for the developmental period between 4 to 9 months gestation. Trabeculae were imaged using µCT and features of their morphology were measured using BoneJ^72^, a plugin for Fiji^73^.

### Specimens

A femur and humerus from 35 fetal specimens of known sex and age were imaged in this study. The specimens were stillbirths and fetal age was documented to the nearest gestational month (4–9) near time of collection, based on maternal testimony (see Table 1). The 35 femora and 35 humeri were osteological specimens from a historical collection held by the Department of Human Anatomy and Cell Biology, Liverpool (McColl et al. 2006). The specimens originated from a Liverpool workhouse and were collected around the beginning of the 20th Century. Specimens had been de-fleshed by bicuspid beetles and stored without further treatment. For a discussion of issues around data collected from historical specimens see Reissis and Abel ^13^.

**Table 1:** Specimen demographics showing the number of male and female fetal specimens (*n*) by gestational month for fetal humeri (n = 35) and femora (n = 35).

Fetal age (months)	Females (*n*)	Males (*n*)	Total (*n*)
4	3	2	5
5	3	4	7
6	3	3	6
7	3	3	6
8	3	3	6
9	2	3	5
Total	17	18	35

During data collection and exchange, all current local policies for use and storage of historical human remains were adhered to. All procedures performed in this study were in accordance with the ethical standards of the institutional and national research committees, and with the 1964 Helsinki Declaration and its later amendments.

### Laboratory µCT and image processing

Specimens were wrapped in X-ray transparent film, mounted in florist foam with their long-axis perpendicular to the beam direction, and scanned using a Nikon Metrology HMX-ST computed tomography cone beam projection system with a tungsten detector panel. Scanning settings were standardised for all specimens (180 kV, 170 µA, 0.1 mm copper filter, 3160 projections were collected through 360°, 16-bit greyscale), and projection data was reconstructed (CTPro, Nikon, Tring, UK) using a modified Feldkamp back-projection algorithm to create 16-bit 3D TIFF image stacks with an isotropic voxel size of 10 µm. The Nyquist-Shannon sampling theorem states a signal (i.e. the width of a trabecula) must be sampled by at least twice the bandwidth of the signal to allow accurate characterisation^32^. This voxel size was deemed sufficient based on prior 2D studies of the proximal femoral metaphysis, which indicated the thinnest trabeculae, which were located closest to the growth cartilage, were 45 ± 10 µm in thickness between 16 and 27 weeks gestation^19^.

Following reconstruction, spherical volumes of interest (VOIs) of trabecular bone were digitally extracted from the 3D TIFF image stacks using VG StudioMax 2.0, processed in Fiji, and digital histomorphometry was performed using the BoneJ plugin. For each fetal specimen, the VOI was the maximal sphere obtainable from the proximal femoral metaphysis immediately inferior to the growth plate without including any cortical bone. Accuracy of VOI was confirmed by examining orthogonal views centred on the VOI and examining the image stage for evidence of cortical bone (see Fig. S1). Comparison of VOI placement in femur and humerus, and at 4 months and 9 months are shown in Fig. S2.

All 3D image stacks underwent a standard processing sequence: (1) application of a 3D median filter (kernel = 1 × 1x1) to all VOIs to reduce image noise and improve quality of binarization^74^; (2) binarization using a global threshold method (the IsoData algorithm of BoneJ) applied to a histogram of the CT grey vales of all voxels in the VOI^75^; (3) “purification” to remove small unconnected regions of foreground voxels, using the *Purification* feature of BoneJ plugin. This is necessary for accurate measurement of connectivity density (see below)^72^. This sequence resulted a binarized single foreground element representing trabecular bone (and background representing marrow space) for each spherical VOI. Following purification, the 3D image stacks were used for digital histomorphometry and 3D volumetric reconstruction.

### Digital histomorphometry

Following processing, the diameter of each VOI was recorded to allow a simple monitoring of growth, and standard digital histomorphometry was performed using the BoneJ plugin to collect standard measures of trabecular morphology: bone volume fraction (BV/TV); mean trabecular thickness (mean Tb.Th); degree of anisotropy (DA); connectivity density (Conn.D); and fractal dimension (FD). The morphometric methods are described in detail elsewhere^72^, but are outlined in brief here.

BV/TV is the volume of bone (i.e., the foreground element) relative to the total volume (TV) of the spherical VOI including the marrow space (i.e., the background element). The BoneJ plugin reports BV as the number of foreground voxels in the image stack (multiplied by voxel size) and TV as the total number of voxels in the image stack (multiplied by the voxel size). As the VOI was spherical (with diameter *d*) but the image stack was cubic (of width *d*) the TV used in BV/TV and Conn.D measures was first corrected by multiplying by a geometric correction factor of 1/6 × pi.

Tb.Th indicates the average 3D thickness of the trabecular bone. It is calculated for every point in the bone by calculating the diameter of the largest spheres that fit within the bone and contain that point^76^. These diameters are then averaged to provide the Tb.Th.

DA is an index of orientation of trabeculae within a VOI, with 0 indicating isotropy (random arrangement) and 1 anisotropy (perfect co-alignment of trabeculae). It was calculated using the “mean intercept length” (MIL) method^77^. This involves generating many vectors from a single randomly placed point. When each vector reaches an interface between bone and marrow, an “intercept” is counted for that vector. For each vector, the MIL is the vector length divided by the number of intercepts. A point cloud is then constructed from each vector multiplied by its MIL, which is then fitted by an ellipsoid, from which eigenvalues related to the length of the ellipsoid axes can be determined. DA is then calculated as: (1—eigenvalue of the longest axis/the eigenvalue of the shortest axis). Following this, the same vectors are sampled at new random points, and DA is modified with new MIL counts, until the coefficient of variation of DA falls below a threshold.

Conn.D is a topological term indicating the degree to which the trabecular architecture is interconnected. It is calculated by dividing the Euler characteristic by the TV^78^. Note this approach assumes a single foreground element, hence the need to employ a *Purify* filter prior to measurement. Trabecular number (Tb.N) was not determined for as it is not directly measured using the BoneJ plugin. Tb.N calculations also require assumptions about the 3D bone geometry (i.e. the structural model)^27^, which are not valid (see comment on structural model index below).

FD is a measure indicating the extent to which trabeculae are self-similar across multiple length-scales^79,80^. This was measured using box-counting algorithm, where increasingly small boxes are scanned over the image and the number of boxes of each size containing the foreground element is counted. As the box size diminishes, the proportion of boxes containing foreground element increases fractally. -log (box size) versus log (box count) is plotted and the FD is the slope of a linear regression line fitted to this.

Finally, although still widely reported, structural model index (SMI) was not calculated as an indicator of rod versus plate morphology of trabeculae^81^, as this approach requires assumptions about the convexity of trabeculae which have been shown to be invalid^82^.

### Data analysis and statistics

To test the effect of sex on fetal trabecular architecture, histomorphometric parameters were compared between females and males for the humerus, femur, or data from both bones combined. To test for site-specific differences in fetal trabecular architecture, parameters were compared between humerus and femur for females, males, or data from both sexes combined. For both sex effects and site-specific differences, comparisons were made using one-way ANOVA with Šídák *posthoc* tests. Finally, to look for changes in fetal trabecular architecture during gestation, parameters obtained from specimens aged at gestational month 4 were compared with those from specimens aged at gestational months 5, 6, 7, 8 and 9 for both humerus and femur. Comparisons for each fetal trabecular architectural parameter were performed using one-way ANOVA with Šídák *posthoc* tests and statistical significance was set at *p* < 0.05*.*

### Supplementary Information

Supplementary Information.

## Data Availability

Raw morphometric data for each volume has been made available as an Excel spreadsheet in Supplementary material (Tables S1 and S2).
